# Enlarged perivascular spaces, neuroinflammation and neurological dysfunction in NMOSD patients

**DOI:** 10.3389/fimmu.2022.966781

**Published:** 2022-09-29

**Authors:** Xiao-Ying Yao, Mei-Chun Gao, Shu-Wei Bai, Li Xie, Ya-Ying Song, Jie Ding, Yi-Fan Wu, Chun-Ran Xue, Yong Hao, Ying Zhang, Yang-Tai Guan

**Affiliations:** ^1^ Department of Neurology, Ren Ji Hospital, School of Medicine, Shanghai Jiao Tong University, Shanghai, China; ^2^ Clinical Research Center, School of Medicine, Shanghai Jiao Tong University, Shanghai, China

**Keywords:** neuromyelitis optica spectrum disorders (not in MeSH), glymphatic circulation, perivascular spaces, neuroinflammation, blood brain barrier (BBB)

## Abstract

**Background and objectives:**

Cerebrospinal fluid (CSF) and interstitial fluid exchange along a brain-wide network of perivascular spaces (PVS) termed the ‘glymphatic system’. The aquaporin-4 (AQP4) water channels abundantly expressed on astrocytic endfeet play a key role in the CSF circulation in the glymphatic system. Neuromyelitis optica spectrum disorder (NMOSD) is an inflammatory demyelinating autoimmune disease of the central nervous system (CNS) featured with a specific autoantibody directed against AQP4 in most of patients. Anti-AQP4 antibodies are likely resulting in the impairment of the brain glymphatic system and the enlargement of PVS in NMOSD patients. In the current study, we aimed to demonstrate the features of EPVS detected by MRI and its association with the CSF anti-AQP4 antibody titer, CNS inflammatory markers, and disease severity in NMOSD patients.

**Methods:**

We conducted a retrospective review of a consecutive cohort of 110 patients with NMOSD who had brain MRI. We assessed the correlation of EPVS with markers of neuroinflammation, blood-brain barrier (BBB) function and severity of neurological dysfunction in patients. We used multivariate logistic regression analysis to determine the independent variables associated with disease severity.

**Results:**

The median number of total-EPVS was 15.5 (IQR, 11-24.2) in NMOSD patients. The number of total-EPVS was significantly related to EDSS score after correcting for the effects of age and hypertension (r=0.353, p<0.001). The number of total-EPVS was also significantly associated with the titer of CSF anti-AQP4 antibody, the albumin rate (CSF/serum ratios of albumin), the CSF albumin, IgG and IgA levels. Logistic regression analysis showed that total-EPVS and serum albumin level were two independent factors to predict disease severity in NMOSD patients (OR=1.053, p=0.028; OR=0.858, p=0.009 respectively). Furthermore, ROC analysis achieved AUC of 0.736 (0.640-0.831, p<0.001) for total-EPVS to determine severe NMOSD (EDSS 4.5-9.5).

**Discussion:**

In our cohort, we found a relationship between EPVS and neuroinflammation and BBB function in NMOSD. Moreover, EPVS might independently predict neurological dysfunction in patients with NMOSD.

## Introduction

The brain was long believed to be devoid of a lymphatic vascular system. In 1970s, Cserr and colleagues has suggested a fluid-transport system in the brain; however, only late in 2012, this lymphatic transport system was designated the glial-associated lymphatic system, or the ‘glymphatic system (1, 2). The glymphatic system facilitates movement of cerebrospinal fluid (CSF) and interstitial fluid (ISF) in the brain (3). Astrocytes play a key role in the glymphatic system. Astrocytes create with their vascular endfeet the perivascular spaces (PVS) that surround the cerebral vasculature. The PVS are utilized as ‘highways’ for fast transport of CSF into deep brain regions (4, 5). The movement of CSF into the parenchyma is facilitated by the aquaporin-4 (AQP4) water channels abundantly expressed on astrocytic endfeet (1, 6). The dysfunction of AQP4 will result in reduced CSF influx into the brain parenchyma and might cause the enlargement of PVS (7). However, there are still some controversies regarding the existence of the glymphatic system, its underlying driving force, and the convective versus diffusive nature of the flow (2).

It has been shown that the glymphatic system plays a role in neuroinflammation and immune responses within the brain (3). And the lymphatic impairment aggravates neuroinflammation probably by the accumulation or entrapment of waste and pro-inflammatory cytokines within the brain (8).

Studies demonstrated that the number and size of PVS may change in the course of inflammatory diseases such as multiple sclerosis (MS) (9–13). Histopathological studies have identified tissue perivascular spaces (EPVS) containing leucocyte infiltrates around chronic active inflammatory lesions of MS (14, 15). These evidence support the role of glymphatic system and pathogenic value of EPVS in neuroinflammatory process.

Neuromyelitis optica spectrum disorder (NMOSD) is an inflammatory demyelinating autoimmune disease of the central nervous system (CNS) mainly affected optic nerve, spinal cord and brain (16). The discovery of an NMO-specific autoantibody directed against AQP4 clearly identified NMOSD as a separate disease from MS (17). Patients can presented with recurrent optic neuritis, relapsing transverse myelitis, and some brainstem and encephalitic syndromes (18).

The autoantibodies that attack the glial AQP4 water channels are likely resulting in the impairment of the brain glymphatic system in NMOSD patients (6). We hypothesize that the gymphatic impairment will cause the enlargement of PVS and the aggravation of neuroinflammation; the latter in turn might lead to more severe neurological dysfunction in NMOSD patients.

So far, the clinical significance of the glymphatic system and EPVS in NMOSD is unknown. We suggested they might play key roles in the immune process of NMOSD. However, glymphatic system is difficult to learn *in situ* while EPVS can be easily visualized on magnetic resonance imaging (MRI). And EPVS could partially reflect the function of glymphatic system. Therefore, in the current study, we aimed to demonstrate the features of EPVS detected by MRI and its association with the CSF anti-AQP4 antibody, CNS inflammatory markers, blood-brain barrier (BBB) function, and disease severity in NMOSD patients.

## Methods

### Study population

The medical records of consecutive patients admitted to our center from April 2013 to January 2022 with the diagnosis of NMOSD were reviewed. Patients who fulfilled the diagnostic criteria established by Wingerchuk et al. in 2015 (19) were included in the current study. The following patients were excluded: (1) those without the head MR images; (2) serum anti-myelin oligodendrocyte glycoprotein (MOG) antibody positive and diagnosed with MOG antibody-associated disease (MOGAD).

### Standard protocol approvals, registrations, and patient consents

The study was approved by the Ethics Committee of RenJi Hospital. Informed consent was obtained from all the included patients.

### Clinical data collection

The clinical, laboratory, and radiology records of all the included patients were retrospectively reviewed. The clinical information collected including age, sex, age at onset, past medical history, total disease duration, time from last relapse, annualized relapse rate (ARR), Expanded Disability Status Scale (EDSS) score, serum status and titers of anti-AQP4 antibody, cerebrospinal fluid results (including white cell counts, albumin, IgG, IgM, IgA, albumin rate, IgG index and anti-AQP4 antibody titer), presentation of optic neuritis (ON), myelitis, and longitudinally extensive transverse myelitis (LETM). The patient was regarded as being in the acute phase of the attack if the time from last relapse was less than 30 days (20). EDSS score was checked at the time of MRI examination. It is used as a scale to evaluate the severity of neurological dysfunction of NMOSD patients. Mild NMOSD was defined with EDSS 0-4.0; while severe NMOSD was defined with EDSS 4.5-9.5. We define the relapse of NMOSD as a clinical exacerbation presenting with new or worsening symptoms accompanied with a change on the neurologic examination that correlated with a new or enhancing MRI lesion. The interval should be at least 30 days since the previous relapse (21). Serum anti-AQP4 and anti-MOG antibodies were tested in all patients using a cell-based assay. Albumin rate is the quotient of (CSF/Ser)*10^-3^, which indicates the disruption of blood-brain barrier.

### MR protocol

Patients underwent MRI according to a standardized protocol as part of routine clinical assessments. The protocol included T1- and T2-weighted, fluid-attenuated inversion recovery (FLAIR), axial trace diffusion-weighted imaging (DWI) with 2 b-values (0 and 1000), and apparent diffusion coefficient (ADC) sequences. All studies were performed on 3.0 T scanners. Images were 2D sequences. Sequences typically included 20–30 slices of 5-mm thickness. The imaging parameters were as follows: T1 (repetition time [TR] 194 ms; echo time [TE] 3.11 ms; field of view [FOV] 200×220; matrix 224×352; pixel 0.9×0.6); T2 (TR 5000 ms; TE 101 ms; FOV 207×220; matrix 224×352; pixel 0.9×0.6); FLAIR (TR 8000 ms; TE 102 ms; FOV 207×220; matrix 203×320; pixel 1.0×0.7); diffusion tensor imaging (TR 3260 ms; TE 50\83 ms; FOV 220×220; matrix 160×160; pixel 1.4×1.4).

### Assessment of EPVS

Enlarged PVS (EPVS) are commonly seen in the centrum semiovale (CSO-EPVS), basal ganglia (BG-EPVS) and midbrain (MB-EPVS) (5).

**Figure 1:**
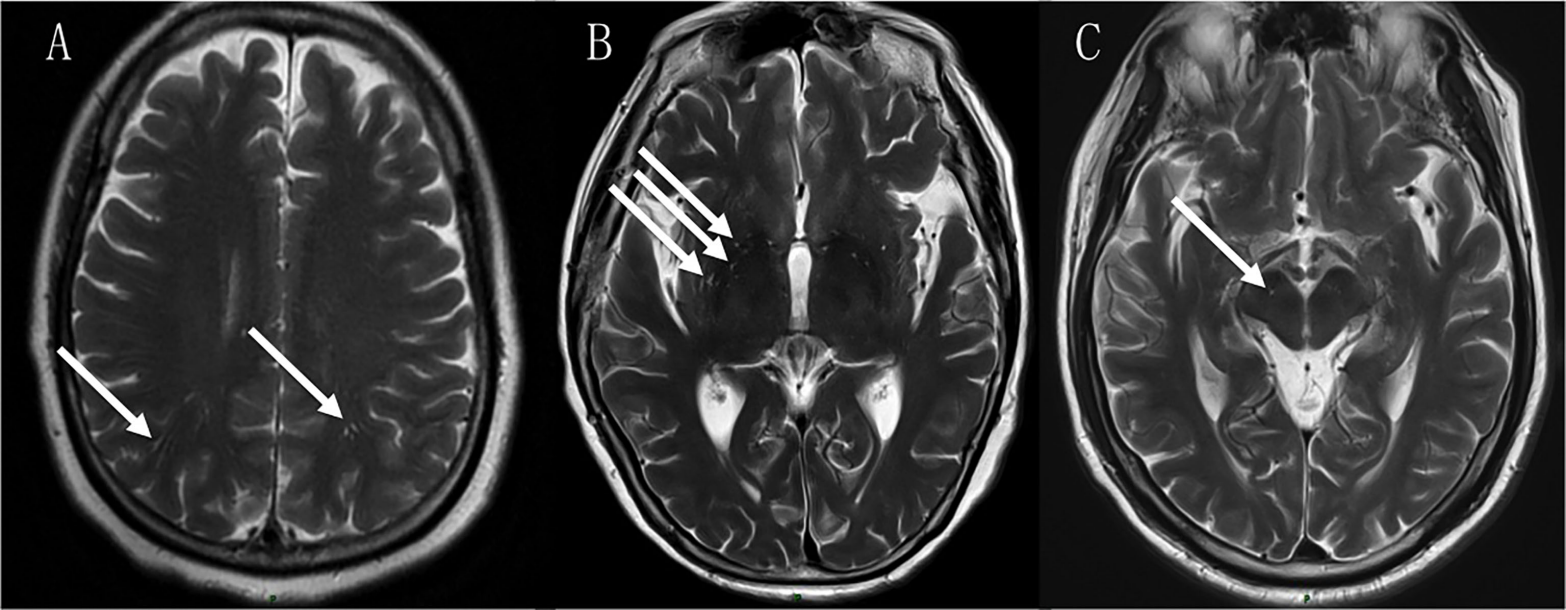
EPVS in the centrum semiovale (CSO-EPVS) **(A)**, basal ganglia (BG-EPVS) **(B)** and midbrain (MB-EPVS) **(C)** in NMOSD patients.

EPVS were rated on axial T2-weighted MRI using a validated visual rating scale (22–24). EPVS were defined as ≤2 mm round or linear CSF isointense lesions (T2-hyperintense and T1/FLAIR hypointense with respect to brain) along the course of penetrating arteries (**Figure 1**). They were distinguished from lacunes by the latter’s large size (>2 and ≤15mm) and surrounding rim of FLAIR hyperintensity (25).

EPVS were separated and rated in the BG, CSO and MB regions. They were counted in both sides on the brain slice showing the greatest extent of EPVS. A total number of EPVS (total-EPVS) was calculated as the sum of EPVS in the three regions.

The following rating categories were used: 0 = no EPVS, 1 = 1 to 10 EPVS, 2 = 11 to 20 EPVS, 3 = 21 to 40 EPVS, and 4 = more than 40 EPVS. For the purpose of this analysis, EPVS were categorized into 0–2 vs 3–4 grades (mild vs severe EVPS).

Two trained neurologists evaluated MRI scans and when inconsistency existed between the reported results both neurologists discussed the case and reached an accord.

### Statistical analysis

Data analysis was performed using SPSS version 21.0 (SPSS Inc., Chicago, Ill., USA). For the statistical analysis on the anti-AQP4 antibody titer X, a logarithmic value of log_2_(X+1) was used. The Kolmogrov-Smirnov Z test was used to verify the normal distribution of the data. Categorical variables were summarized as counts (percentage) and continuous variables as the means (standard deviation, SD) or medians (interquartile ranges, IQR), if not distributed normally. Statistical comparisons between two groups were performed using Student’s t-test or the Mann–Whitney U test for continuous variables, as well as the χ2 and Fisher’s exact tests for categorical variables, as deemed appropriate. Correlations between continuous variables were assessed by Spearman’s correlation coefficient. Logistic regression analysis was used to detect the independent factors associated with severe EPVS (EPVS grade 3-4) or severe NMOSD (EDSS 4.5-9.5). Receiver operating characteristic (ROC) curve and area under curve (AUC) were used to test the accuracy for EPVS to determine disease severity. A two-tailed probability value < 0.05 was considered significant. We did not correct for multiple comparisons.

## Results

### Clinical data of patients with NMOSD

As **Table 1** shows, a total of 110 patients with NMOSD were enrolled. The mean age was 48.1 years (range 15-83 years); the female patients (n=93) had a dominant percentage of 84.5%; 67 (60.9%) patients were in the acute phase. The serum anti-AQP4 antibody was positive in 93 (84.5%) patients. CSF anti-AQP4 antibody was tested in 72 patients and 46 (63.9%) were positive. The annualized relapse rate (ARR) was 0.5 (IQR 0-1.1). The median score of EDSS was 3.5 (IQR, 2.5-6.1) at the time of MRI examination. Thirty patients were diagnosed comorbidities of autoimmune disorders (including Sjogren’s syndrome, systemic lupus erythematosus, rheumatoid arthritis, mixed connective tissue disease, ankylosing spondylitis, and hashimoto thyroiditis). And the rates of hypertension, diabetes mellitus, and hyperlipidemia were 17.3%, 12.7% and 10.9% respectively.

**Table 1:** Clinical data of patients with neuromyelitis optica spectrum disorders (NMOSD).

	NMOSD (N=110)			
	AllN=110	EPVS grade 0-2N=71	EPVS grade 3-4N=39	P value
**Age** (mean ± SD)	48.1 ± 14.4	43.6 ± 12.9	56.1 ± 13.4	<0.001
**Sex** (Female, %)	93 (84.5%)	60 (84.5%)	33 (84.6)	0.988
**Age at onset** (mean ± SD)	44.3 ± 15.7	39.8 ± 13.8	52.5 ± 15.9	<0.001
**Acute phase (%)**	67 (60.9%)	41 (57.7%)	26 (66.7%)	0.359
**Total disease duration** (median, IQR, months)	19.5 (2.8-68.2)	23.0 (3.0-72.0)	14.0 (2.0-67.0)	0.597
**Time from last relapse** **(**median, IQR, days**)**	15 (7-60)	21 (7-60)	15 (7-37)	0.338
**Numbers of all attacks** (median, IQR)	2 (1-3)	2 (1-3)	2 (1-4)	0.730
**Annualized relapse rate (ARR)** (median, IQR)	0.5 (0-1.1)	0.4 (0-1.0)	0.5 (0-2.0)	0.424
**Co-morbidities**				
**Other Autoimmune Disorders** (%)	30 (27.3%)	22 (31.0%)	8 (20.5%)	0.238
**Systemic lupus erythematosus** (%)	6 (5.5%)	5 (7.0%)	1 (2.6%)	0.420
**Hypertension (%)**	19 (17.3%)	8 (11.3%)	11 (28.2%)	0.025
**Diabetes Mellitus (%)**	14 (12.7%)	6 (8.5%)	8 (20.5%)	0.129
**Hyperlipidemia (%)**	12 (10.9%)	8 (11.3%)	4 (10.3%)	1.000
**History of ischemic stroke or** **Transient ischemic attack (%)**	2 (1.8%)	1 (1.4%)	1 (2.6%)	1.000
**History of smoking (%)**	6 (5.5%)	5 (7.0%)	1 (2.6%)	0.582
**History of alcoholism (%)**	1 (0.9%)	0 (0%)	1 (2.6%)	0.355
**Clinical presentations**				
ON ^a^ (%)	50 (45.5%)	31 (43.7%)	19 (48.7%)	0.610
Myelitis (%)	84 (76.4%)	53 (74.6%)	31 (79.5%)	0.568
LETM ^b^ (%)	70 (63.6%)	42 (59.2%)	28 (71.8%)	0.187
CSF^d^ analysis				
**White cell counts** (median, IQR)	2.0 (0-7.0)	2.0 (0-7.2)	2.0 (0-6.5)	0.500
**Albumin** (mg/L, median, IQR)	344 (217-432)	322 (184-394)	398 (302-528)	0.023
Albumin rate ^e^ (median, IQR**)**	5.8 (4.0-8.9)	5.0 (3.7-7.8)	6.6 (5.4-10.1)	0.008
**IgG (**mg/L, median, IQR**)**	35.7 (24.1-57.0)	30.2 (22.6-54.8)	43.4 (29.8-75.7)	0.028
**IgA (**mg/L, median, IQR**)**	3.9 (2.6-7.2)	3.5 (1.9-7.3)	5.2 (3.0-7.2)	0.062
**IgM (**mg/L, median, IQR**)**	0.6 (0.3-1.2)	0.6 (0.2-1.0)	0.5 (0.3-1.3)	0.764
**IgG index** (median, IQR)	0.52 (0.47-0.59)	0.51 (0.47-0.58)	0.54 (0.47-0.59)	0.310
Anti-AQP4 antibody ^f^ (%)	46 (63.9%)	26 (56.5%)	20 (76.9%)	0.083
**Titer of Anti-AQP4 (log_2_)** ^g^ (median, IQR)	1.0 (0-2.1)	1.0 (0-2.1)	2.1 (0.75-3.5)	0.033
**Serum Anti-AQP4 antibody** (%)	93 (84.5%)	58 (81.7%)	35 (89.7%)	0.264
**Titer of serum Anti-AQP4 (log_2_)** ^h^ (median, IQR)	5.9 (0-8.3)	5.0 (0-8.3)	6.7 (3.5-8.3)	0.599
**Serum Albumin (g/L,** mean ± SD**)**	41.2 ± 4.3	42.0 ± 4.1	39.8 ± 4.5	0.01
EDSS ^i^ (median, IQR)	3.5 (2.5-6.1)	3.0 (2.0-4.0)	6.0 (3.0-8.0)	<0.001
EPVS counts ^j^				
BG-EPVS ^k^	7.0 (5.0-11.0)	6.0 (4.0-8.0)	11.0 (9.0-15.0)	<0.001
CSO-EPVS ^l^	8.0 (4.0-12.2)	5.0 (3.0-8.0)	18.0 (13.0-22.0)	<0.001
**MB-EPVS** ^m^	0 (0-1.0)	0 (0-1.0)	1.0 (0-2.0)	0.001
**Total-EPVS**	15.5 (11-24.2)	13 (8-15)	29 (24-37)	<0.001

a ON, optic neuritis.

b LETM, longitudinally extensive transverse myelitis.

c Brain syndromes of NMOSD refer to area postrema syndrome, brainstem syndrome, diencephalic syndrome and cerebral syndrome.

d CSF, cerebrospinal fluid.

e Albumin rate = Quotient (CSF/Ser)*10^-3^.

f Anti-AQP4 antibody, anti-aquaporin-4 antibody.

g For the analysis of the CSF anti-AQP4 antibody titer X, a logarithmic value of log_2_ (X+1) was used.

h For the analysis of the serum anti-AQP4 antibody titer X, a logarithmic value of log_2_ (X+1) was used.

i EDSS, Expanded Disability Status Scale.

j EPVS, enlarged perivascular spaces.

k CSO, centrum semiovale.

l BG, basal ganglia.

m MB, Midbrain.

### The features of EPVS in patients with NMOSD

The median numbers of BG-EPVS, CSO-EPVS and MB-EPVS were 7.0 (IQR, 5.0-11.0), 8.0 (IQR, 4.0-12.2) and 0 (IQR, 0-1.0) respectively. The median number of total-EPVS was 15.5 (IQR, 11-24.2) in NMOSD patients.

We define EPVS grade 0-2 as mild category of EPVS and grade 3-4 as severe category of EPVS. There were 39 (35.5%) patients had severe EPVS. NMOSD patients with severe EPVS as compared to those with mild EVPS were older (56.1 ± 13.4 vs 43.6 ± 12.9, p<0.001) and had a higher percentage of hypertension (28.2% vs 11.3%, p=0.025). Severe EPVS patients tended to have higher levels of CSF albumin and CSF IgG, higher titers of CSF anti-AQP4 antibody, and a lower level of serum albumin. The median albumin rate was higher in patients with severe EPVS, which indicates a more severe blood-brain barrier disruption. Moreover, EDSS score was significantly higher in severe EPVS group as compared to mild EPVS group (3.0 vs 6.0, p<0.001). (**Table 1**) And there were more numbers of total-EPVS in patients with CSF anti-AQP4 antibody positive than negative (18.5 vs 13.0, p=0.034).

Sensitivity analyses were conducted by comparing lowest EPVS group (EVPS grade 0-1) with highest EPVS group (EPVS grade 4) and similar results were shown. (**Supplementary materials: Table 1**)

### Correlation of EPVS with markers of neuroinflammation, blood-brain barrier function and severity of neurological dysfunction in NMOSD patients

**Figure 2:**
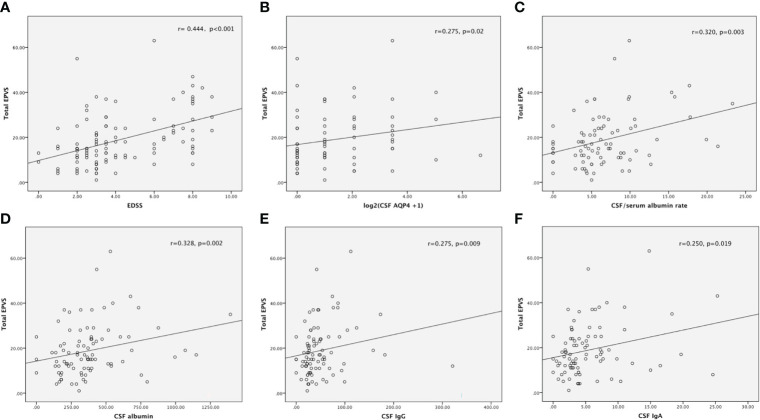
The number of total-EPVS was significantly correlated with EDSS score **(A)**, CSF titer of anti-AQP4 antibody **(B)**, CSF/Serum albumin rate **(C)**, the levels of CSF albumin **(D)**, CSF IgG **(E)** and CSF IgA **(F)**.

The number of total-EPVS was significantly correlated with EDSS score (r= 0.444, p<0.001), which means NMOSD patients with more numbers of EPVS had more severe neurological dysfunction. (**Figure 2A**) The positive relationship between numbers of total-EPVS and EDSS scores still existed when stratified patients by age, serum anti-AQP4 status, disease phase, and first attack or relapse. (**Supplementary materials: Table 2**)

Previous studies have highlighted associations between EPVS severity and increasing age and vascular risk factors such as hypertension (26). Therefore, in order to exclude the influence of age and hypertension, partial correlation analysis was performed. The result revealed that the number of total-EPVS was still significantly related to EDSS score after correcting for the effects of age and hypertension (r=0.353, p<0.001).

For different areas of EPVS, the numbers of CSO-, BG-, and MB- EPVS were respectively associated with EDSS score (r=0.352, p<0.001 for CSO; r=0.454, p<0.001 for BG; r=0.200, p=0.036 for MB). However, after correcting for the effects of age and hypertension, only the positive relationship between the numbers of CSO-, BG- EPVS and EDSS score remained significant (r=0.265, p=0.006 for CSO; r=0.385, p<0.001 for BG).

The number of total-EPVS was significantly associated with the titer of CSF, but not serum, anti-AQP4 antibody (r=0.275, p=0.02). (**Figure 2B**) For different areas of EPVS, only the number of CSO-EPVS was significantly related to the titer of CSF anti-AQP4 antibody (r=0.254, p=0.031).

Moreover, the number of total-EPVS was significantly correlated to the albumin rate (r=0.320, p=0.003), which indicates NMOSD patients with more numbers of EPVS were accompanied with more serious blood-brain barrier disruption. (**Figure 2C**) And both CSO-EPVS and BG-EPVS were related to the albumin rate (r=0.239, p=0.028 for CSO-EPVS; r=0.396, p<0.001 for BG-EPVS).

Furthermore, total-EPVS was significantly associated with CSF albumin, IgG and IgA levels (r=0.328, p=0.002 for CSF albumin; r=0.275, p=0.009 for CSF IgG; r=0.250, p=0.019 for CSF IgA), which implied that more numbers of total-EPVS were related to more severe central nervous inflammation. (**Figures 2D-F**)

### Logistic analysis of independent factors associated with severe EPVS in NMOSD patients

Two logistic regression models were conducted to find out the independent factors associated with severe EPVS (EPVS grade 3-4). As **Table 2** showed, age and EDSS were independent factors in model 1 (including the covariates of age, history of hypertension and EDSS); while age and CSF titer of anti-AQP4 antibody were independent factors in model 2 (including the covariates of age, history of hypertension, CSF titer of anti-AQP4 antibody, CSF IgG, CSF IgA, CSF/serum albumin rate, serum albumin and EDSS). (**Table 2**)

**Table 2:** Evaluation of independent factors associated with severe EPVS ^a^ (grade 3-4) in patients with neuromyelitis optica spectrum disorders by logistic regression analysis.

Model 1				
Variates	B	Exp(B)/OR	95% confidence interval	P value
**Age (years)**	0.058	1.059	1.017-1.103	0.005
History of Hypertension	0.366	1.442	0.415-5.008	0.564
**EDSS** ^b^	0.298	1.347	1.107-1.639	0.003
**Model 2**				
**Variates**	**B**	**Exp(B)/OR**	**95% confidence interval**	**P value**
**Age (years)**	0.097	1.102	1.033-1.175	0.003
History of Hypertension	0.983	2.672	0.541-13.201	0.228
CSF titer of Anti-AQP4 ^c^	0.559	1.748	1.046-2.922	0.033
CSF IgG **(**mg/L)	-0.029	0.971	0.936-1.008	0.127
CSF IgA **(**mg/L)	-0.039	0.971	0.798-1.159	0.679
CSF/Serum albumin rate ^d^	0.205	1.228	0.949-1.590	0.119
Serum Albumin (g/L)	0.046	1.047	0.879-1.246	0.607
EDSS	0.054	1.056	0.686-1.626	0.806

a EPVS, enlarged perivascular spaces.

b EDSS, Expanded Disability Status Scale.

c For the analysis of the CSF anti-AQP4 antibody titer X, a logarithmic value of log_2_(X+1) was used.

d Albumin rate = Quotient (CSF/Ser)*10^-3^.

### Logistic analysis of independent predictors of disease severity in NMOSD patients

**Figure 3:**
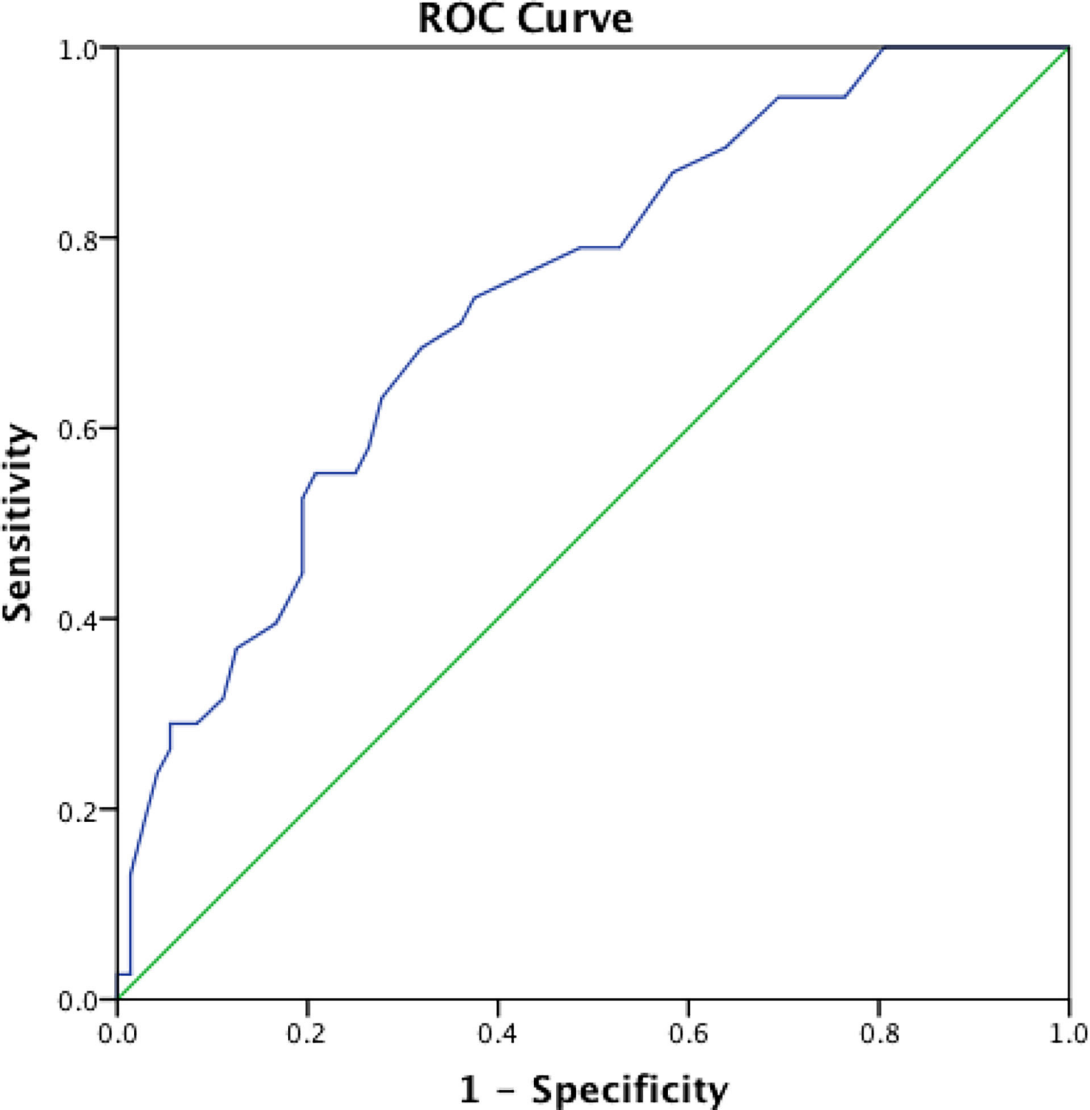
The ROC curve for total-EPVS to determine severe NMOSD (EDSS 4.5-9.5).

In order to clarify the independent predictive effect of total-EPVS with disease severity in NMOSD, patients were divided into two groups: mild NMOSD group (EDSS 0-4.0) (N=72) and severe NMOSD group (EDSS 4.5-9.5) (N=38). The variates of total-EPVS, as well as age, age of onset, numbers of all attacks, and serum albumin were entered into the logistic model. The result showed that total-EPVS and serum albumin level were two independent factors in the model to predict disease severity (OR=1.053, 95%CI 1.006-1.102, p=0.028 for total-EPVS; OR=0.858, 95% CI 0.765-0.962, p=0.009 for serum albumin) (**Table 3**). Furthermore, ROC analysis achieved AUC of 0.736 (0.640-0.831, p<0.001) for total-EPVS to determine severe NMOSD. (**Figure 3**)

**Table 3:** Evaluation of independent predictors of severe neuromyelitis optica spectrum disorders (EDSS 4.5-9.5) by logistic regression analysis.

Variates	B	Exp(B)/OR	95% confidence interval	P value
Age (years)	-0.034	0.967	0.841-1.112	0.637
Age of onset (years)	0.054	1.056	0.923-1.207	0.429
**Serum Albumin (g/L)**	-0.153	0.858	0.765-0.962	0.009
Number of all attacks	0.077	1.080	0.871-1.340	0.483
**Total-EPVS**	0.051	1.053	1.006-1.102	0.028

EDSS, Expanded Disability Status Scale.

EPVS, enlarged perivascular spaces.

## Discussion

Our study demonstrated that more total-EPVS was significantly correlated with higher CSF anti-AQP4 antibody titer, more severe blood-brain barrier disruption and intenser neuroinflammation. Moreover, total-EPVS was an independent predictor of severe neurological dysfunction in NMOSD.

PVS is part of the structure of glymphatic system. Studies have already proved that PVS are important in immune responses and inflammatory processes within the brain (12, 27). EPVS are believed to be associated with blood-brain barrier leakage and associated infiltration of monocytes, lymphocytes, and macrophages in the PVS (28).

Pathological examination showed abundant antibody-secreting cells were noted in perivascular spaces in autoimmune glial fibrillary acidic protein (GFAP) astrocytopathy, a CNS inflammatory disease targeting astrocytes (29). In a case report of Behçet’s disease, the neuropathological examination revealed an acute inflammation consisted of a neutrophilic and eosinophilic infiltration of the perivascular spaces and brain parenchyma (30). It is suggested that during the pathogenesis of MS, T cell activation begins at the periphery of the lymphoid compartment (extracerebral area) and then reaches the CNS, with the T cells circulating in the PVS (10). In an animal model of progressive MS, immunohistological analysis showed that mature and isotype-switched B cells predominately localized to the meninges and perivascular space, with IgG isotype-switched B cells frequently accumulating in the parenchymal space (31).

Astrocytic endfeet and their dense expression of the aquaporin-4 water channels promote fluid exchange between the perivascular spaces and the neuropil. Thus, anti-AQP4 antibody targeting the main water channels on asctrocytes will impair CSF influx from PVS to neuropil in NMOSD (6). Therefore, we presume that the glymphatic system and EPVS might play even more important roles in the pathogenesis of NMOSD than the above-mentioned CNS inflammatory diseases.

Our results showed that patients with higher CSF titer of anti-AQP4 antibody had more EPVS on MRI. Higher level of CSF antibody will impair more water channels on astrocyte endfeet of PVS, which in turn block the influx of CSF and lead to the enlargement of PVS.

We also found that EPVS are associated with neuroinflammation and BBB disruption in NMOSD. As our data showed, severe EPVS are related to higher CSF albumin, IgG and IgA levels, which implied an intense inflammatory reaction in CNS; severe EPVS are also correlated with a greater impairment of BBB indicated by higher CSF/serum ratios for albumin. It has already been proved that glymphatic impairment aggravates CNS inflammation by suppressing cytokine clearance from the brain (8). The BBB is a very efficient barrier formed by the vascular endothelial cells, their tight junctions, and the underlying basement membrane. The BBB can be considered the internal boundary of PVS at the capillary level (32). Therefore, inflammation in the PVS can aggravate the impairment of BBB, and vice versa. It still remains unclear as to whether PVS dilation is a cause, effect, or secondary process of endothelial dysfunction and increased BBB permeability (33). Previous studies also demonstrated EPVS to be a marker of BBB dysfunction (34), as well as a marker of neuroinflammation (10).

Our study also demonstrated that EPVS was independently associated with neurological dysfunction in NMOSD. Since EPVS can indicate inflammation in CNS, thereby more EPVS are related to more severe clinical presentation. Previous studies in MS have already demonstrated that increased numbers of EPVS are associated with clinical disability (23, 35).

The results of the current study prompt us to raise the following hypothesis. Anti-AQP4 antibodies originate in peripheral and enter the PVS through endothelial transcytosis or at areas of increased BBB permeability. Then anti-AQP4 antibodies in PVS bind selectively to AQP4 on astrocyte endfeet. This interaction results in down-regulation of surface AQP4 and less clearance of waste and pro-inflammatory cytokines in the CNS. Moreover, it activates complement produced locally by astrocytes, which in turn leads to increased BBB permeability and massive infiltration of leukocytes and cytokines. And inflammation in PVS will increase the numbers and volumes of EPVS. The neurological dysfunction will also aggravate in the condition of more severe neuroinflammation.

There are several limitations in the current study. One limitation is the relatively small number of patients included and the retrospective design of the study; although the statistical analyses were significant, the correlations were relatively weak. Therefore, large-scale prospective studies should be conducted with longer periods of follow-up to confirm the results drawn from the current study. Secondly, more biomarkers of neuroinflammation and BBB function such as CSF pro-inflammatory cytokines and radiological studies should be used to further prove the current results. Thirdly, the glymphatic system has been proved to be existed in brain and optic nerves; however, whether it exists in spinal cord need further studies. It is an important question to be explored because spinal cord is an end organ frequently attacked in NMOSD. Finally, we also found a relationship between EVPS and disease severity in anti-AQP4 antibody negative NMOSD patients and it could not be explained by the above-mentioned hypothesis. There might be other mechanisms involved.

## Conclusion

In conclusion, we found a relationship between EPVS and neuroinflammation and BBB function in NMOSD. Moreover, EPVS might independently predict neurological dysfunction in patients with NMOSD.

## Data availability statement

The raw data supporting the conclusions of this article will be made available by the authors, without undue reservation.

## Ethics statement

The studies involving human participants were reviewed and approved by ethics committee of Ren Ji Hospital. The patients/participants provided their written informed consent to participate in this study.

## Author contributions

X-YY: conception, methodology, data analysis, and writing original draft. M-CG: resources, data collection. S-WB: resources, data collection. LX: data analysis. Y-YS: resources, data collection. C-RX: resources, data collection. Y-FW: resources, data collection. YH: data curation. YZ: validation, methodology. Y-TG: conception. supervision, writing – review and editing. All authors contributed to the article and approved the submitted version.

## Funding

This study was supported by the National Natural Science Foundation of China (No. 81801211) and the Outstanding Youth Training Funds of Shanghai Ren Ji Hospital (No. PYII-17-001). Innovative research team of high-level local universities in Shanghai (SHSMU-ZDCX20211901).

## Conflict of interest

The authors declare that the research was conducted in the absence of any commercial or financial relationships that could be construed as a potential conflict of interest.

## Publisher’s note

All claims expressed in this article are solely those of the authors and do not necessarily represent those of their affiliated organizations, or those of the publisher, the editors and the reviewers. Any product that may be evaluated in this article, or claim that may be made by its manufacturer, is not guaranteed or endorsed by the publisher.
