# Supplementary material for: Enlarged perivascular spaces, neuroinflammation and neurological dysfunction in NMOSD patients
Source: Front Immunol. 2022 Sep 29;13:966781. doi: 10.3389/fimmu.2022.966781 (PMC9557144; doi:10.3389/fimmu.2022.966781)
Supplement: Supplementary file 1 [file Table_1.docx]

Supplementary Materials

Table 1 Sensitivity analysis by comparing lowest EPVS group (EVPS scores 0-1) with highest EPVS group (EPVS score 4) in patients with neuromyelitis optica spectrum disorders

|  | **NMOSD（N=29）** | | | |
| --- | --- | --- | --- | --- |
|  | **All**  **N=29** | **EPVS grade 0-1**  **N=24** | **EPVS grade 4**  **N=5** | **P value** |
| **Age** (median, IQR) | 39.0(30.5-50.0) | 37.0(29.2-46.5) | 70(51-79.5) | **0.001** |
| **Sex** (Female, %) | 23(79.3%) | 19(79.2%) | 4(80%) | 0.731 |
| **Age at onset** (median, IQR) | 37.0(26.0-46.5) | 34.5(25.5-42.0) | 69(39-75) | **0.032** |
| **Acute phase (%)** | 16(55.2%) | 14(58.3%) | 2(40%) | 0.396 |
| **Total disease duration**  (median, IQR, months) | 34.0(14.0-73.5) | 35(14-75) | 30(9.5-177.5) | 0.889 |
| **Time from last relapse**  **(**median, IQR, days**)** | 20(8-75) | 17.5(7.5-52.5) | 58(11-1352) | 0.201 |
| **Numbers of all attacks**  (median, IQR) | 3(2-4) | 3(2-3.75) | 3(1.5-7.5) | 0.634 |
| **Annualized relapse rate (ARR)**  (median, IQR) | 0.4(0.1-0.8) | 0.4(0.1-0.7) | 0.3(0.1-1.7) | 0.933 |
| **Co-morbidities** |  |  |  |  |
| **Other Autoimmune Disorders** (%) | 6(20.7%) | 5(20.8%) | 1(20%) | 0.731 |
| **Systemic lupus erythematosus** (%) | 1 (3.4%) | 1 (4.2%) | 0 (0%) | 0.828 |
| **Hypertension (%)** | 5(17.2%) | 3(12.5%) | 2(40%) | 0.195 |
| **Diabetes Mellitus (%)** | 4(13.8%) | 2(8.3%) | 2(40%) | 0.127 |
| **Hyperlipidemia (%)** | 2(6.9%) | 1(4.2%) | 1(20%) | 0.320 |
| **History of ischemic stroke or**  **Transient ischemic attack (%)** | 1(3.4%) | 0(0%) | 1(20%) | 0.172 |
| **History of smoking (%)** | 2(6.9%) | 2(8.3%) | 0(0%) | 0.680 |
| **History of alcoholism (%)** | 0(0%) | 0(0%) | 0(0%) | - |
| **Clinical presentations** |  |  |  |  |
| ON ^a^ (%) | 13(44.8%) | 10(41.7%) | 3(60%) | 0.396 |
| Myelitis (%) | 22(75.9%) | 18(75%) | 4(80%) | 0.653 |
| LETM ^b^ (%) | 18(62.1%) | 14(58.3%) | 4(80%) | 0.356 |
| **CSF^d^ analysis** |  |  |  |  |
| **White cell counts** (median, IQR) | 1.5(0-3.8) | 1(0-3) | 3(0-27) | 0.616 |
| **Albumin** (mg/L, median, IQR) | 314(176-398) | 287(175-353) | 531(433-676) | **0.03** |
| **Albumin rate ^e^ (**median, IQR**)** | 5.0(3.8-9.2) | 5.0(3.8-6.6) | 9.9(8.0-17.7) | **0.03** |
| **IgG (**mg/L, median, IQR**)** | 30.8(23.5-65.3) | 28.3(23.3-55.1) | 74.2(42.1-112) | 0.053 |
| **IgA (**mg/L, median, IQR**)** | 3.9(2.2-9.4) | 3.9(1.8-6.8) | 14.8(5.4-25.3) | **0.040** |
| **IgM (**mg/L, median, IQR**)** | 0.45 (0.20-2.14) | 0.4(0.2-2.0) | 1.3(0.4-14.8) | 0.226 |
| **IgG index** (median, IQR) | 0.51(0.48-0.60) | 0.51(0.47-0.59) | 0.55(0.51-0.66) | 0.308 |
| **Anti-AQP4 antibody ^f^** (%) | 9(45%) | 7(43.8%) | 2(50%) | 0.625 |
| **Titer of Anti-AQP4 (log_2_)** ^g^  (median, IQR) | 0(0-2.1) | 0(0-2.1) | 1.0(0-3.1) | 0.750 |
| **Serum Anti-AQP4 antibody** (%) | 23(79.3%) | 18(75%) | 5(100%) | 0.283 |
| **Titer of serum Anti-AQP4 (log_2_)** ^h^  (median, IQR) | 5.4(0-9.1) | 5.0(0-9.6) | 3.5(1.7-9.1) | 0.845 |
| **Serum Albumin (g/L,** median, IQR**)** | 41.9(39.4-44.8) | 42.1(39.7-45.3) | 41.8(34.6-42.4) | 0.222 |
| **EDSS ^i^** (median, IQR) | 3.0 (2.0-3.75) | 3.0(2.0-3.0) | 8.0(4.0-8.25) | **0.023** |

^a^ ON: optic neuritis

^b^ LETM: longitudinally extensive transverse myelitis

^c^ Brain syndromes of NMOSD refer to area postrema syndrome, brainstem syndrome, diencephalic syndrome and cerebral syndrome

^d^ CSF: cerebrospinal fluid

^e^ Albumin rate = Quotient (CSF/Ser)*10^-3^

^f^ Anti-AQP4 antibody: anti-aquaporin-4 antibody

^g^ For the analysis of the CSF anti-AQP4 antibody titer X, a logarithmic value of log_2_(X+1) was used

^h^ For the analysis of the serum anti-AQP4 antibody titer X, a logarithmic value of log_2_(X+1) was used

^i^ EDSS: Expanded Disability Status Scale

^j^ EPVS: enlarged perivascular spaces

^k^ CSO: centrum semiovale

^l^ BG: basal ganglia

^m^ MB: Midbrain

Table 2 Correlation between numbers of total-EPVS and EDSS scores in NMOSD patients.

| **Characteristics** | **n** | **Total-**  **EPVS ^a^**  (median, IQR) | **EDSS ^b^**  (median, IQR) | **r** | **P value** |
| --- | --- | --- | --- | --- | --- |
| **Total** | 110 |  |  | 0.444 | ＜0.001 |
| **Age** |  |  |  |  |  |
| Age≥45 | 62 | 19.5（13.8-28） | 3.5（3.0-6.6） | 0.367 | 0.003 |
| Age＜45 | 48 | 13（8-18） | 3.0（2.0-6.0） | 0.392 | 0.006 |
| **Anti-AQP4 antibody ^c^** |  |  |  |  |  |
| Positive | 93 | 17（11.5-25） | 3.5（2.5-6.0） | 0.414 | ＜0.001 |
| Negative | 17 | 15（8.5-20.5） | 3.0（2.0-7.0） | 0.528 | 0.029 |
| **First attack/relapse** |  |  |  |  |  |
| First attack | 28 | 16.5（11.5-25） | 3.5（2.0-6.0） | 0.562 | 0.002 |
| Relapse | 82 | 15（11-24） | 3.5（2.5-6.6） | 0.407 | ＜0.001 |
| **Disease phase** |  |  |  |  |  |
| Acute phase | 67 | 17（11-25） | 3.5（3.0-7.0） | 0.470 | ＜0.001 |
| Chronic phase | 43 | 15（11-24） | 3.0（2.0-4.5） | 0.433 | 0.004 |

^a^ EDSS: Expanded Disability Status Scale

^b^ EPVS: enlarged perivascular spaces

^c^ Anti-AQP4 antibody: anti-aquaporin-4 antibody
